# Hysterectomy for postpartum hemorrhage in Japan: Diagnostic code validation and nationwide descriptive analysis

**DOI:** 10.1111/jog.70019

**Published:** 2025-07-28

**Authors:** Eishin Nakamura, Tadahiro Goto, Shigetaka Matsunaga, Akihiko Kikuchi, Yasushi Takai, Sayuri Shimizu

**Affiliations:** ^1^ Center for Maternal, Fetal and Neonatal Medicine Saitama Medical Center, Saitama Medical University Saitama Japan; ^2^ Department of Health Data Science Yokohama City University Yokohama Japan; ^3^ TXP Medical Co. Ltd., Tokyo Japan; ^4^ Department of Clinical Epidemiology and Health Economics, School of Public Health The University of Tokyo Tokyo Japan; ^5^ Department of Obstetrics and Gynecology, Saitama Medical Center Saitama Medical University Saitama Japan

**Keywords:** health services research, hysterectomy, maternal mortality, national database, postpartum hemorrhage

## Abstract

**Aim:**

Hysterectomy is a life‐saving procedure for severe postpartum hemorrhage (PPH), but reports on postoperative mortality are limited. This study aimed to describe the rates of hysterectomy and associated mortality in PPH patients using the Diagnosis Procedure Combination (DPC) database, Japan's largest inpatient database.

**Method:**

We first validated the accuracy of PPH diagnostic coding at a tertiary perinatal center, then conducted a nationwide descriptive analysis using DPC data from April 2018 to March 2023. The DPC database includes over half of all acute care hospital admissions in Japan. PPH cases were identified using International Statistical Classification of Diseases and Related Health Problems, 10th Revision codes and blood loss data. We examined hysterectomy rates and postoperative mortality, including a subgroup excluding cases with conditions requiring planned hysterectomy during cesarean section (e.g., placenta previa, accreta, uterine rupture, and cervical cancer).

**Results:**

The validation study showed high accuracy of PPH coding, with a sensitivity of 97.8% and specificity of 99.7%. Among 209 555 PPH cases, 1835 (0.88%) underwent hysterectomy, with a mortality rate of 0.87% (16 deaths). After excluding 23 039 cases with indications for planned hysterectomy, 681 of 186 516 cases (0.36%) required hysterectomy, with a higher mortality rate of 2.2%.

**Conclusions:**

The DPC database reliably identifies PPH cases. Hysterectomy was performed in 0.88% of all PPH cases, with higher mortality in emergency cases after excluding planned procedures.

## INTRODUCTION

Postpartum hemorrhage (PPH) is a common complication of all deliveries and is one of the leading causes of maternal death.^1^ PPH requires immediate intervention because the disease progresses rapidly after onset, and hemostasis is difficult when the patient is in a hemorrhagic state. The treatment of PPH consists primarily of medical treatment (e.g., blood transfusions and coagulation factor replacement), uterine artery embolization, and surgical interventions. The choice of treatment depends on the patient's condition and the primary source of bleeding. Among surgical options, hysterectomy is the most invasive procedure and serves as the definitive hemostatic measure when other minimally invasive procedures fail.

The indications for hysterectomy to achieve hemostasis in patients with PPH remain unclear. Surveillance of PPH cases across the United States reported that hysterectomy was performed in 0.9% of all PPH patients.^2^ Similarly, Pettersen et al. reported that hysterectomy was performed in 1.6% of patients with severe PPH.^3^ In contrast, a Japanese study by Ueda et al. reported a higher hysterectomy rate of 6.4% among patients with severe PPH (defined as blood loss ≥500 mL for vaginal delivery and ≥1000 mL for cesarean section).^4^ However, the Japanese study did not analyze postoperative mortality or causative diseases of hysterectomy for PPH, nor did it verify the accuracy of the data utilized. Furthermore, a few reports in the literature provide accurate and comprehensive data regarding aspects. Obtaining precise information on the rate of hysterectomy and its associated mortality in Japan is crucial for reevaluating the foundational treatment strategies for PPH.

As described previously, research on hysterectomy for PPH is limited. This is likely due to the limited number of studies that have effectively analyzed medical databases and the lack of validation of results in large‐scale database studies. To address these gaps, the present study was conducted in two phases.

1. A validation study of PPH coding accuracy and outcomes using single‐center data.
2. A nationwide descriptive analysis was conducted using the Diagnosis Procedure Combination (DPC) database to examine hysterectomies for PPH, including causative conditions, surgery rates, and mortality rates.

We first validated the accuracy of the disease codes registered in the DPC database by comparing them with the medical records. The DPC database, the largest medical database in the country, is specifically designed for acute care hospitals and provides comprehensive data on inpatient care. Using this database, we then conducted a nationwide descriptive analysis of hysterectomies for PPH in Japan, focusing on the causative conditions, surgery rates, and associated mortality rates.

## MATERIALS AND METHODS

### Validation of coding accuracy of PPH and outcomes

#### Study design and setting

This study aimed to validate the accuracy of administrative codes in identifying patients with PPH (bleeding volume ≥1000 mL). We conducted a diagnostic accuracy analysis using medical records from the Saitama Medical Center, Saitama Medical University, covering the period from April 1, 2018, to March 31, 2022. As the largest perinatal hospital in Saitama Prefecture, the center provides comprehensive treatment for PPH and handles approximately 1000 deliveries annually. It also serves as the largest tertiary care facility in the region, receiving approximately 40–50 PPH patient transfers annually. Additionally, the center has a team of approximately 20 obstetricians and gynecologists specializing in perinatal care. This validation study was conducted after obtaining approval from the Ethics Committee of Saitama Medical Center, Saitama Medical University (Ethics Committee approval number: 2022‐117).

To assess the accuracy of PPH case identification in the DPC database, we extracted data for all patients discharged from our institution between April 1, 2018, and March 31, 2023, who met the PPH criteria based on both medical records and the DPC database. PPH cases were identified using the following criteria: (i) the presence of any disease names listed in Table S1, and (ii) recorded blood loss exceeding 1000 mL during delivery. Criterion (i) primarily identified PPH cases associated with coagulopathy, while criterion (ii) followed the American College of Obstetricians and Gynecologists (ACOG) definition, which considers a blood loss volume of ≥1000 mL as a key diagnostic indicator of PPH^5^. Treating medical records as the gold standard, we constructed a cross‐tabulation table to determine true positives, false positives, false negatives, and true negatives for PPH cases identified in the DPC database.

### Descriptive analysis of hysterectomy for PPH using DPC data

#### Participants

This study included patients who required inpatient management due to a diagnosis of PPH between April 1, 2018, and March 31, 2023. PPH cases were identified based on the following criteria:

- Presence of the disease names listed in Table S1.
- Blood loss exceeding 1000 mL during delivery.

For criterion (i), patients with PPH were primarily identified as having coagulopathy, including those diagnosed with intrapartum hemorrhage, unspecified (International Statistical Classification of Diseases and Related Health Problems, 10th Revision [ICD‐10] code: O679), other immediate PPHs (O721), and postpartum coagulation defects (O723). Criterion (ii) was based on the ACOG definition of PPH, which uses a blood loss volume of 1000 mL or more as a key indicator.^5^

The DPC database does not capture blood loss data from delivery facilities before patient transfer, making it difficult to identify severe PPH cases referred from primary to tertiary care centers. To address this limitation and minimize false negatives, we combined ICD‐10 disease codes with delivery blood loss data, defining PPH as cases meeting at least one of the following criteria: (i) having any of the aforementioned ICD‐10 codes or (ii) experiencing blood loss exceeding 1000 mL during delivery.

#### Data source

The database used in this study was independently developed by a research group that solicited data provision from medical institutions participating in the Ministry of Health, Labor and Welfare's (MHLW) annual “Survey on the Impact of DPC Introduction.” Unlike the DPC data routinely submitted to the MHLW, this research database consisted of data voluntarily provided by individual hospitals with written consent for research purposes.

The DPC database, originally introduced in 2003 as a nationwide inpatient database supporting comprehensive reimbursement for acute hospitalizations.^6^ By 2021, the database included 44 million admissions and discharges from 1332 acute care hospitals, representing more than half of Japan's acute care patients.^7^ The DPC database collects detailed information on patient demographics, diagnoses at admission, comorbidities, procedures, medications, and in‐hospital adverse events.

Diagnoses, comorbidities, and adverse events were categorized according to the ICD‐10, while surgeries and procedures were recorded using unique 9‐digit electronic receipt processing codes. The database captures key clinical information, including in‐hospital deaths, surgeries and procedures, discharge outcomes, and blood loss during delivery.

This study utilizing the DPC database was approved by the Ethics Committee of the Tokyo Medical and Dental University (Approval Number: M2000‐788).

#### Collected variables

From the eligible patients, we extracted data on age, blood loss at delivery, comorbid conditions—including placenta previa, placenta accreta, and uterine rupture—interventional procedures such as hysterectomy and/or arterial embolization, and mortality outcomes.

To identify the patients who underwent total hysterectomy or arterial embolization for PPH, we used the procedure codes listed in the DPC database. Patients who underwent hysterectomy or arterial embolization were identified using the procedure codes (as listed in Table S1) recorded within 7 days of admission. Facilities were classified as capable of arterial embolization if they had undergone at least one such procedure during the study period.

To identify patients who underwent therapeutic hysterectomy for PPH more accurately, we extracted comorbidities that could lead to hysterectomy based on the comorbidity names recorded in the database. These comorbidities were identified using ICD‐10 codes and included placenta previa (O441), adherent placenta (O432, O720, O722, O730), uterine rupture (O710, O711), and cervical cancer (C530, C531, C538, C539).

Additionally, cases without any comorbid conditions were categorized as having an “absence of any uterine or placental comorbid conditions.” These patients were assumed to have undergone hysterectomy solely for hemostasis, likely due to conditions such as atonic uterine bleeding. Death was defined as death occurring during the same hospitalization period.

#### Statistical analyses

All statistical analyses were performed using R software (version 4.3.1). Patients who underwent a hysterectomy were classified into the hysterectomy group, whereas those who did not undergo a hysterectomy were classified into the non‐hysterectomy group. Continuous variables were compared using the Wilcoxon rank‐sum test, while categorical variables were compared using Fisher's exact test. A *p*‐value of less than 0.05 was considered statistically significant.

## RESULTS

### Validation study

**FIGURE 1:**
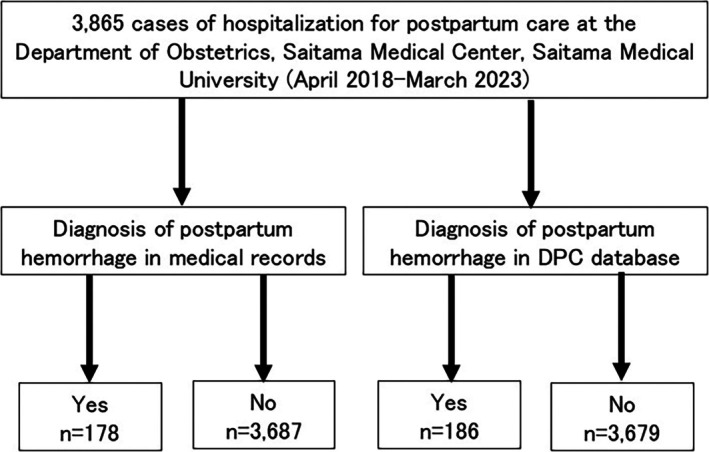
Patient flow diagram for the validation study.

Figure 1 shows the patient flow chart for the validation study. From 2018 to 2023, 3865 patients were hospitalized for postpartum care at the Department of Obstetrics of Saitama Medical Center, Saitama Medical University. Of these, 178 patients were diagnosed with PPH, defined as blood loss ≥1,000 mL in the medical record. Analysis of the in‐hospital DPC data during the same period identified 186 PPH cases. Table 1 shows the cross‐tabulation of PPH diagnoses in the DPC data, using medical records as the gold standard. The diagnostic validity of PPH in the DPC data was exceptionally high, with a sensitivity of 97.8%, specificity of 99.7%, a positive predictive value of 93.5%, and a negative predictive value of 99.9%.

**TABLE 1:** Confusion matrix of disease names in medical records and DPC data.

		Postpartum hemorrhage patient diagnosed from medical records		
		Yes	No	
Postpartum hemorrhage patient diagnosed from DPC data	Yes	174	12	186
	No	4	3675	3679
		178	3687	3865

Abbreviation: DPC, Diagnosis Procedure Combination.

### Analysis of the DPC database

**FIGURE 2:**
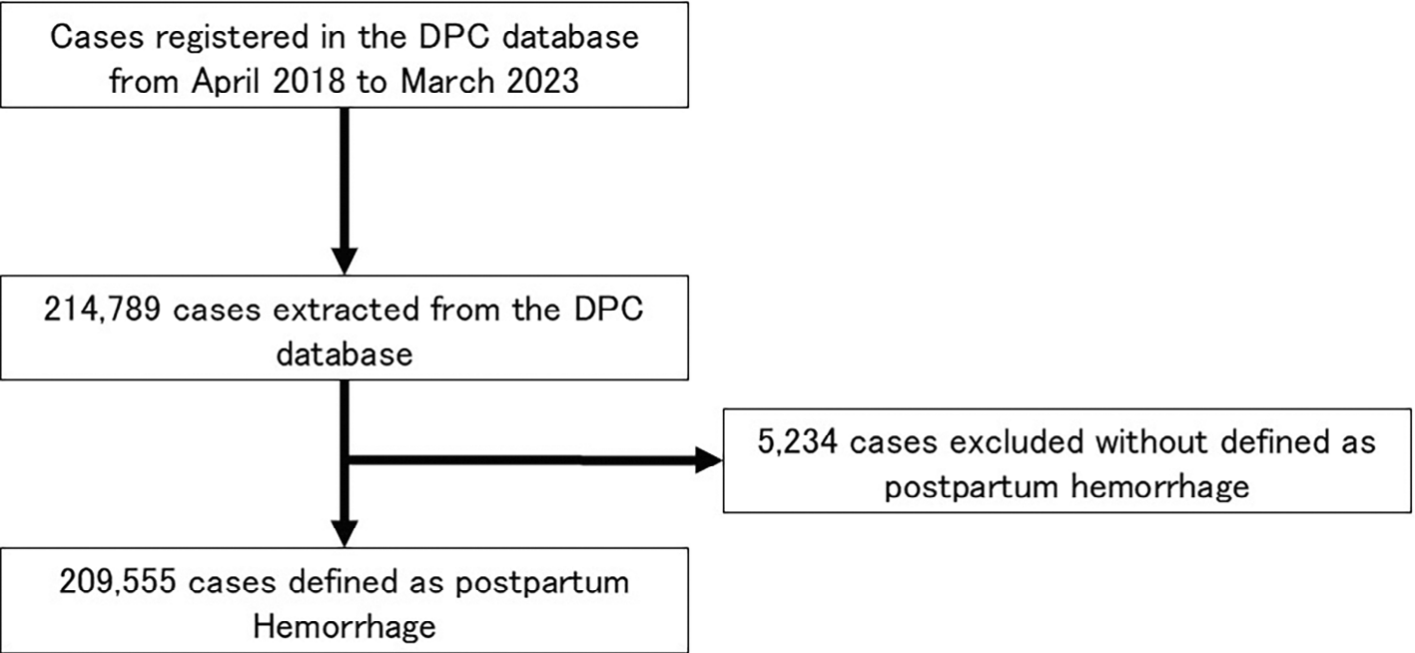
Patient flow diagram for analysis of the DPC database. DPC, Diagnosis Procedure Combination.

Figure 2 shows a diagram of the patient flow. The data obtained from the DPC database from 2018 to 2023 included 214 789 patients. Of these, 5234 cases that did not meet the definition of PPH were excluded, leaving 209 555 cases for the analysis.

Table 2 presents the characteristics of the 209 555 patients with PPH included in the analysis. Of these, 1835 patients (0.88%) underwent hysterectomy. A total of 155 685 patients (74.3%) were treated at institutions where embolization was available, and uterine artery embolization was performed in 3511 patients (1.68%). There were 103 deaths (0.05%, 49.2 per 100 000). The table also compares the patient characteristics between those who did and did not undergo hysterectomy, showing significant differences between the groups across all categories except for the proportion of cervical cancer‐related comorbidities. The hysterectomy group demonstrated significantly higher rates of comorbid conditions, including placenta accreta and placenta previa, and a higher mortality rate (0.87% vs. 0.04%).

**TABLE 2:** Patient characteristics and comparison by hysterectomy status.

	Overall (*n* = 209 555)	Hysterectomy group (*n* = 1835)	Non‐hysterectomy group (*n* = 207 720)	*p*‐value
Maternal age (years, mean ± SD)	33.51 ± 5.38	33.48 ± 5.38	36.36 ± 4.78	<0.001*
Cesarean delivery (%)	124 157 (59.2%)	785 (42.8%)	123 372 (59.4%)	<0.001*
Treatment at facilities capable of arterial embolization (%)	155 685 (74.3%)	1647 (89.8%)	154 038 (74.2%)	<0.001*
Comorbid conditions				
Placenta accreta (%)	8797 (4.20%)	731 (39.8%)	8066 (3.88%)	<0.001*
Placenta previa (%)	14 612 (6.97%)	601 (32.8%)	14 011 (6.75%)	<0.001*
Uterine rupture (%)	333 (0.16%)	141 (7.68%)	192 (0.09%)	<0.001*
Cervical cancer (%)	312 (0.15%)	8 (0.44%)	304 (0.15%)	0.004*
Clinical progress				
Bleeding during delivery (ml, mean ± SD)	1330.2 ± 698.9	3125 ± 1908	1315 ± 660	<0.001*
Uterine artery embolization (%)	3511 (1.68%)	332 (18.1%)	3179 (1.53%)	<0.001*
Hysterectomy (%)	1835 (0.88%)	1835	0	—
Length of hospital stay (days, mean ± SD)	12.3 ± 12.8	22.12 ± 18.82	12.22 ± 12.65	<0.001*
Death (%)	103 (0.05%)	16 (0.87%)	87 (0.04%)	<0.001*

Abbreviations: SD, standard deviation.

* *p*‐values were calculated using Fisher's exact test for categorical variables and the Wilcoxon rank‐sum test or *t*‐test for continuous variables, as appropriate.

Finally, we selected patients without any comorbid conditions requiring hysterectomy during or after delivery. After excluding 23 039 patients with comorbid conditions, the remaining 186 516 patients were analyzed. Among them, 681 (0.36%) required hysterectomy, with a mortality rate of 2.2%. The results remained consistent, with significantly higher mortality in the hysterectomy group (2.20% vs. 0.05%; Table 3).

**TABLE 3:** Patient characteristics classified by whether or not a hysterectomy was performed, only for patients without comorbid conditions that require hysterectomy as an intervention.

	Hysterectomy group (*n* = 681)	Non‐hysterectomy group (*n* = 185 835)	*p*‐value
Maternal age (mean ± SD)	36.20 ± 5.27	33.36 ± 5.42	<0.001*
Cesarean delivery (%)	240 (35.2%)	108 536 (58.4%)	<0.001*
Treatment at facilities capable of arterial embolization (%)	596 (87.5%)	135 844 (73.1%)	<0.001*
Clinical progress			
Bleeding during delivery (mL, mean ± SD)	3098 ± 1912	1272 ± 625	<0.001*
Uterine artery embolization (%)	120 (17.6%)	2080 (1.12%)	<0.001*
Length of hospital stay (days, mean ± SD)	16.90 ± 15.70	11.61 ± 11.94	<0.001*
Death (%)	15 (2.20%)	86 (0.05%)	<0.001*

Abbreviations: SD: standard deviation.

* *p*‐values were calculated using Fisher's exact test for categorical variables and the Wilcoxon rank‐sum test or *t*‐test for continuous variables, as appropriate.

## DISCUSSION

This study evaluated the accuracy of the DPC database in identifying PPH cases and provided nationwide estimates of hysterectomy rates and postoperative mortality among patients with PPH. The validation study demonstrated that the combination of ICD‐10 codes and recorded blood loss accurately identified PPH cases, with high sensitivity (97.8%) and specificity (99.7%). Nationwide analysis using the validated method showed that 0.88% of the patients with PPH underwent hysterectomy, with an associated mortality rate of 0.87%. After excluding cases with comorbid conditions necessitating planned hysterectomy, the hysterectomy rate among remaining patients with PPH was 0.36%, with a mortality rate of 2.2%. The mortality rates were significantly higher in the hysterectomy group than in the non‐hysterectomy group.

Previous studies have reported varying rates of hysterectomy for PPH, influenced by differences in healthcare systems, institutional protocols, and the availability of alternative interventions such as uterine artery embolization. While hysterectomy rates in Japan appear to be lower than those reported in other countries, our findings align with those of previous Japanese studies, suggesting a more conservative approach to surgical management. The mortality rate in the hysterectomy group was higher than that in Japan, reflecting the severity of cases requiring this intervention. The observed difference in mortality rates between patients undergoing planned hysterectomies for conditions such as placenta previa and those requiring emergency hysterectomies highlights the impact of preoperative management strategies on patient outcomes. Hysterectomy for PPH is typically performed when hemostasis cannot be achieved using other methods and is deemed necessary to save the mother's life. Damage Control Surgery has been established in the emergency departments as a treatment strategy for patients with trauma. In recent years, PPH patients have been managed according to this principle.^8^ In this study, the mortality rate of the hysterectomy group (0.9%, 872 per 100 000 deliveries) was higher than the maternal mortality rate in Japan (0.0025%, 2.5 per 100 000 deliveries),^9^ which may be partly attributed to the invasive nature of the surgical procedure. Furthermore, the higher mortality rate in the hysterectomy group may reflect the inclusion of more severely ill patients who underwent hysterectomy.

This study provides robust evidence for the validity of the DPC database for identifying PPH cases, which can facilitate future epidemiological research and quality improvement initiatives. The findings highlight the importance of early recognition and intervention in PPH to reduce the need for hysterectomy and improve survival outcomes. The significantly higher mortality rate in patients undergoing emergency hysterectomy underscores the need to optimize nonsurgical hemostatic strategies and enhance preparedness for severe PPH cases in clinical settings. In Japan, 45.8% of all births occur in general medical clinics, where PPH can occur. In most cases, PPH in these clinics is managed by transferring patients via ambulance to higher‐level facilities, such as maternal life‐saving control centers.^10^ Given the low institutional volume of hysterectomies for PPH in Japan, efforts to centralize expertise and improve multidisciplinary collaboration may improve patient outcomes.

Future research should focus on refining the patient selection criteria for hysterectomy in PPH, identifying factors influencing mortality beyond hysterectomy status, and evaluating the impact of alternative interventions. Prospective cohort studies or randomized controlled trials are needed to establish the causality between hysterectomy and mortality while accounting for confounding factors such as parity, prior uterine surgeries, and institution‐specific treatment protocols. Further investigation of institutional differences in PPH management and their impact on patient outcomes would also be valuable.

A key strength of this study is that it provided insights into the current status of hysterectomy for PPH, supported by a validation study that confirmed the appropriateness of using the DPC database for PPH research. Although the DPC database relies on ICD‐10 codes for disease classification, there is a possibility of misclassification if case identification is based solely on these codes.^11^ In this study, we incorporated both diagnostic codes and blood loss volume at delivery to identify PPH cases more accurately. This validation study demonstrated the diagnostic accuracy of this approach, supporting the feasibility of using the DPC database for PPH research. Another strength of this study is its comprehensive analysis of both hysterectomy rates and postoperative mortality in patients with PPH. By excluding patients with primary conditions that would typically require a hysterectomy, such as placenta previa and uterine rupture, this study provides a clearer understanding of the risks and outcomes associated with hysterectomy in the context of PPH alone. Another strength is the study's focus on postoperative mortality, which is a crucial, yet often underreported aspect of hysterectomy for PPH. Mortality data derived from a large cohort offers valuable insights into the risks of hysterectomy, contributing to the limited body of research on postoperative outcomes in patients with PPH.

A limitation of this study is that it did not allow us to estimate the treatment effect of hysterectomy on mortality in patients with PPH; therefore, causal inferences regarding the effect of hysterectomy on mortality cannot be made. Potential confounding factors between the hysterectomy and non‐hysterectomy groups may include parity and details of previous treatments; however, this information is not available in the primary large‐scale DPC databases, which are the primary large‐scale databases in Japan. Additionally, the number of hysterectomies performed per institution for PPH in Japan is extremely low compared to that in other countries because of the incomplete consolidation of maternity facilities. In Japan, where the consolidation of maternity facilities lags behind that in Western countries, the number of total hysterectomies per facility remains very low.^12^ Therefore, a multicenter study is needed to make accurate causal inferences regarding the effect of hysterectomy on mortality. Furthermore, the descriptive nature of the current study did not allow for the identification of specific factors that may influence mortality after hysterectomy. Additionally, the mortality rate reported in this study is limited to in‐hospital mortality and does not include long‐term prognosis or deaths occurring after hospital discharge.

This study confirmed the validity of the DPC database for PPH research and provided crucial epidemiological insights into hysterectomy and mortality rates among patients with PPH in Japan. These findings underscore the importance of early intervention and multidisciplinary approaches in managing severe PPH. Further research is needed to refine clinical decision‐making for hysterectomy and evaluate strategies for improving maternal outcomes in high‐risk cases.

## AUTHOR CONTRIBUTIONS

**Eishin Nakamura:** Conceptualization; methodology; software; data curation; investigation; validation; formal analysis; funding acquisition; visualization; project administration; resources; writing – original draft; writing – review and editing. **Tadahiro Goto:** Conceptualization; methodology; software; investigation; validation; formal analysis; supervision; funding acquisition; visualization; project administration; resources; writing – review and editing. **Shigetaka Matsunaga:** Conceptualization; formal analysis; project administration; supervision; writing – review and editing. **Akihiko Kikuchi:** Conceptualization; supervision; writing – review and editing. **Yasushi Takai:** Conceptualization; funding acquisition; project administration; supervision; writing – review and editing. **Sayuri Shimizu:** Conceptualization; data curation; methodology; software; investigation; validation; formal analysis; supervision; funding acquisition; visualization; project administration; resources; writing – review and editing.

## CONFLICT OF INTEREST STATEMENT

The authors declare no conflicts of interest. Dr. Yasushi Takai is an Editorial Board member of the Journal of Obstetrics and Gynaecology Research and a co‐author of this article. To minimize bias, he was excluded from all editorial decision‐making related to the acceptance of this manuscript.

## Supporting information


**Table S1.** Definitions and codes for each disease and procedure in the DPC data.

## Data Availability

The data that support the findings of this study are derived from the Diagnosis Procedure Combination (DPC) database, which is managed by the Ministry of Health, Labour and Welfare of Japan. Due to legal and ethical restrictions, access to the data is limited to authorized researchers and cannot be shared publicly. Data are available upon reasonable request for researchers who meet the criteria for access to confidential data through appropriate institutional review and permission processes.
