# Supplementary material for: Hysterectomy for postpartum hemorrhage in Japan: Diagnostic code validation and nationwide descriptive analysis
Source: J Obstet Gynaecol Res. 2025 Jul 28;51(8):e70019. doi: 10.1111/jog.70019 (PMC12582897; doi:10.1111/jog.70019)
Supplement: Supplementary file 1 — Table S1. Definitions and codes for each disease and procedure in the DPC data. [file JOG-51-0-s001.docx]

*Supplementary Table 1: Definitions and codes for each disease and procedure in the DPC data*

| **Disease name** | **Code** |
| --- | --- |
| *Definition of postpartum hemorrhage* | *ICD-10 code* |
| Intrapartum hemorrhage, unspecified | O679 |
| Other immediate postpartum hemorrhage | O721 |
| Postpartum coagulation defects | O723 |
| *Definition of hysterectomy* | *Receipt electronic processing codes* |
| total hysterectomy | 150217510 |
| supracervical hysterectomy | 150217410 |
| total hysterectomy for uterine rupture | 150222810 |
| supracervical hysterectomy for uterine rupture | 150222910 |
| Total hysterectomy after cesarean section (Porro's operation) | 150223110 |
| *Definition of arterial embolization* | *Receipt electronic processing codes* |
| arterial embolization (hemostasis) | 150360610 |
| arterial embolization (other) | 150360710 |

DPC, Diagnosis Procedure Combination
